# Defining health equity: A modern US perspective

**DOI:** 10.1016/j.isci.2024.111326

**Published:** 2024-11-05

**Authors:** Kristen R. Prentice, Marie Beitelshees, Andrew Hill, Charles H. Jones

**Affiliations:** 1 Department of Exceptional Student Education, University of South Florida, Tampa, FL, USA; 2 Bulmore Consulting, Lockport, NY 14094, USA; 3 Pfizer Inc, 66 Hudson Boulevard, New York, NY 10001, USA

**Keywords:** Health sciences, Public health, Sociology

## Abstract

Health equity is a concept that has gained increasing attention and relevance in the context of the COVID-19 pandemic, which has exposed and exacerbated the health disparities and inequities among different population groups in the United States. This article aims to provide a comprehensive and critical overview of the historical, theoretical, and empirical foundations of health equity, as well as the challenges and opportunities for advancing it in the modern US society. By adopting an interdisciplinary and intersectional approach, and by drawing on literature from public health, sociology, economics, and human rights, we argue that health equity is not only a matter of fairness and justice, but also a strategic and pragmatic goal for improving the health and well-being of the entire nation. Here, we propose a modern definition of health equity for the US context, and conclude with some recommendations for policy, practice, and research to promote health equity in the US

## Graphical abstract

**Figure:**
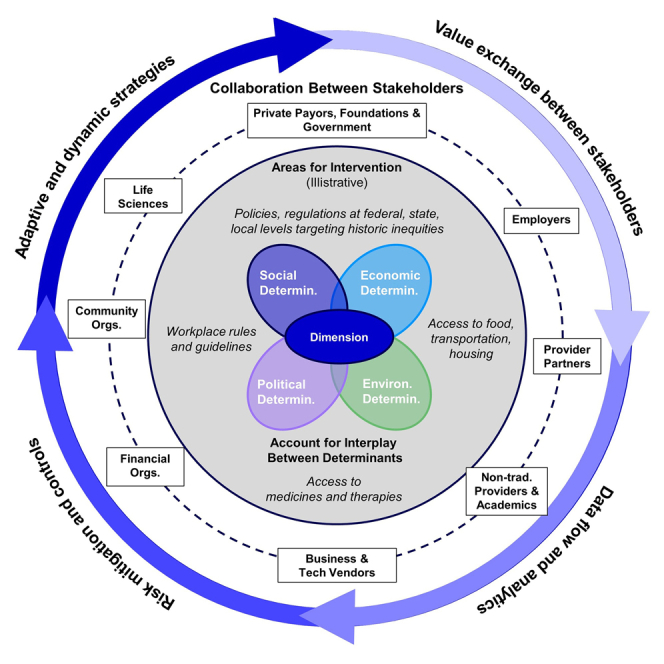

## Introduction

In the United States, the right to quality health remains a contentious and unresolved issue, as healthcare is not universally guaranteed as a human right. Instead, access to healthcare and the quality of that care represents a key indicator of social and economic development that varies significantly across different population groups, reflecting deeply rooted inequities.^1^ Health equity, defined by the World Health Organization as “the absence of avoidable or remediable differences among groups of people,”^2^ remains an aspirational goal rather than a realized state in the US.

While health inequities exist globally, there is a wealth of robust data on health equity in the US, making it an ideal case study for analyzing the impacts of disparities across diverse population groups. The financial capacity of the US to enact substantial policy changes aimed at enhancing equity is also largely unmatched. Despite this, the US consistently ranks the last of the high-income countries on measures of health equity, particularly on financial barriers to accessing and paying for care, difficulty in obtaining care at convenient times, and in patient-reported experiences.^3^

The pursuit of health equity in the US is intertwined with the history of social justice, civil rights, and human rights movements, which have challenged and transformed the structures and systems of oppression, discrimination, and marginalization that have shaped the health and well-being of different population groups in the US (Table 1). While significant strides have been made toward health equity in this country, the long and complex legacy of racial, ethnic, gender, class, and other forms of inequality and injustice have resulted in persistent and pervasive health disparities and inequities among various groups. These groups include, but are not limited to, Black Americans, Native Americans, Latinos, Asian Americans, women, LGBTQ+ people, people with disabilities, immigrants, refugees, and people from low-income households. Such groups have faced, and continue to face, multiple and intersecting barriers to accessing quality and affordable health care, education, employment, housing, transportation, and other social and environmental resources and opportunities that affect their health.^4^^,^^5^ Moreover, these groups have also experienced, and continue to experience, various forms of violence, trauma, stigma, and discrimination that affect their mental, emotional, and physical health.^6^^,^^7^ While this article will provide examples of experiences within groups, a detailed analysis of health inequities across all marginalized groups is not within the scope of this paper. We acknowledge that the experiences discussed here are not universal across all demographics.

**Table 1:** Events in history that influence health equity in the United States (not exhaustive)

Historical event	Dates	Impact on health equity	References
Establishment of the PHS	1798	Marked the beginning of organized medical and public health efforts by the federal government, laying groundwork for future public health initiatives.	CDC^8^
Freedmen’s Bureau	1865–1872	Provided healthcare and education to freed slaves, marking an early effort to address health disparities post-Civil War.	Benjamin et al.^9^
Social Security Act	1935	Established a safety net for the elderly, the poor, and the sick, indirectly affecting health equity by reducing poverty-related health disparities.	Martin et al.^10^
Tuskegee Syphilis Study	1932–1972	Involved experimentation on Black men without their informed consent, leading to long-term mistrust in the healthcare system among Black communities.	Freimuth et al.^11^
Hill-Burton Act	1946	Funded hospital construction across the country, with a requirement for hospitals to provide care to those unable to pay, aiming to increase access.	Thomas et al.^12^
Henrietta Lacks’ HeLa Cells	1951	Cells taken without consent from a Black woman, Henrietta Lacks, leading to significant medical advances but also raising ethical issues about consent and benefit sharing.	Skloot et al.^13^
Establishment of Medicare and Medicaid	1965	Created to provide health insurance to people aged 65 and older and to low-income families, respectively, significantly impacting health equity.	Berkowitz et al.^14^
Forced Sterilization of Native American Women	1970s	Thousands of Native American women were sterilized without their knowledge or consent, significantly impacting their reproductive rights and autonomy.	Torpy et al.^15^
Section 504 of the Rehabilitation Act	1973	Prohibited discrimination on the basis of disability in programs and activities receiving federal financial assistance, enhancing access to services and promoting health equity for individuals with disabilities.	Georgia Tech^16^
National Health Planning and Resources Development Act	1974	Aimed to control healthcare costs and ensure equitable distribution of health resources, impacting health planning and equity.	^17^
Establishment of the ORWH at the NIH	1990	Established to enhance research related to diseases, disorders, and conditions that affect women.	NIH^18^
HIV/AIDS Epidemic and Initial Government Response	Early 1980s	Slow response to the HIV/AIDS epidemic, particularly impacting gay men, intravenous drug users, and the Haitian community, exacerbating stigma and health disparities.	Francis et al.^19^
Ryan White CARE Act	1990	Provided funding for HIV/AIDS treatment for those without sufficient health care coverage or financial resources, addressing a critical health disparity.	Cahill et al.^20^
ADA	1990	Prohibited discrimination against individuals with disabilities in all areas of public life, including jobs, schools, transportation, and all public and private places that are open to the general public, promoting health equity for people with disabilities.	US Department of Labor^21^
WHI	Launched in 1991	One of the largest US prevention studies of its kind, focusing on strategies for preventing heart disease, breast and colorectal cancer, and osteoporotic fractures in postmenopausal women.	NIH^22^
FDA Policy Change on Women in Clinical Trials	1993	Reversed a 1977 policy, now requiring women to be included in clinical drug trials, aiming to ensure that research findings would be applicable to women as well as men.	NIH^18^
Patient Protection and ACA	2010	Expanded healthcare coverage, including to those with pre-existing conditions, aiming to reduce health disparities.	Manchikanti et al.^23^
COVID-19 Pandemic	2020-Present	Disproportionately affected minority and low-income communities, highlighting existing health disparities in disease burden, access to care, and outcomes.	Bambra et al.^24^
George Floyd	2020	His death ignited global protests against racial injustice and police brutality, leading to increased awareness and discussions on systemic racism’s impact on health equity, prompting initiatives to address these disparities.	Fine et al.^25^

ADA, Americans with Disabilities Act; ACA, Affordable Care Act; NIH, National Institute of Health; ORWH, Office of Research on Women’s Health; PHS, Public Health Service; VAWA, Violence Against Women Act; WHI, Women’s Health Initiative.

Red = negative impact on health equity; green = positive impact on health equity.

This table is not exhaustive but provides a starting point for understanding how health equity in the US has been shaped over time.

Several recent events and trends have reignited the discussion around health equity. The stark disparities in health outcomes during the COVID-19 pandemic, for example, have exposed flaws in our current approach to health equity. In fact, numerous studies have demonstrated the unequal burden (e.g., rates of infection, hospitalization, and mortality) borne by groups facing health disparities and inequities during the pandemic.^26^^,^^27^^,^^28^^,^^29^^,^^30^ As a result of this major public health emergency, it has become evident that our approach falls short in addressing the complex interplay of structural inequalities that shape health outcomes. Attempts to provide care for the aging US population has also exposed inequities, particularly in poor and/or rural areas.^31^ The death of George Floyd has also catalyzed renewed focus on racial dynamics and health equity, sparking global protests and awareness about systemic racism’s impact on health.^25^ However, achieving equity transcends individual racial events, requiring a comprehensive approach that addresses the broader population’s diverse needs and systemic disparities.

In fact, what we need is a paradigm shift—an evolution toward a comprehensive framework for health equity that goes beyond conventional boundaries and addresses the underlying causes of health discrepancies. To this end and in this work, we provide an examination of the theoretical and empirical foundations of health equity, focusing on the social, economic, environmental, and political contexts that are shaping the health equity landscape in the US. By adopting an interdisciplinary and intersectional approach, we propose a modern framework for defining health equity, tailored to the US context, with the aim of inspiring policy, practice, and research efforts that address the challenges highlighted by the COVID-19 pandemic and paving the way for a more equitable future. While focusing solely on the US is a limitation in our assessment, many of the ideas we present are universal. As such, the proposed modern definition of health equity should be viewed as a framework that can be adjusted to reflect the unique conditions and challenges faced in each country.

### Current definitions of health equity

The dynamic and multidimensional nature of health equity means that it is particularly difficult to define in an actionable, reliable, and meaningful way. Stakeholders within the healthcare ecosystem all have similar definitions that include common principles: the recognition of the existence and extent of health disparities and inequities among different groups; the identification of the causes and consequences of health disparities and inequities; the commitment to reduce or eliminate health disparities and inequities; and the promotion of fairness, justice, and human rights in health (Table 2).^32^^,^^33^ However, within each stakeholder category, these principles are consistently applied through the lens of their experience, remit, and they are often tied to business objectives.

**Table 2:** Health equity definitions

Across the ecosystem, most stakeholders leverage the CDC Healthy People definition of health equity …		… but when discussing health equity, stakeholders have a narrow focus on what is within their remit	
*CDC*	“Health equity is the state in which everyone has a fair and just opportunity to attain their highest level of health”^34^	*Governments*	“Advancing equity is not just about the many issues we’re addressing, it’s also about the way we do business, which is why our Equity Action Plan focuses on grants, acquisitions, and capacity building” – *HHS*^35^
*Kaiser Family Foundation*	“Health equity generally refers to individuals achieving their highest level of health through the elimination of disparities in health and health care”^36^	*Payers*	“We know that health inequities are very, very costly … Health disparities in the US cost us about $93B in excess medical costs every year” – *Humana Chief Health Equity Officer*^37^
*UnitedHealth Group*	“Health equity is achieved when every person, regardless of race, place or circumstance, has the opportunity to live their healthiest life”^38^	*Providers*	“Creating medical facilities that are welcoming and accessible, to researching and understanding different conditions that affect different populations, to addressing systemic biases that can affect the care delivered to different communities of color” – *Kaiser Permanente*^39^
Common Themes: • Most stakeholders discuss an “attainment of the highest level of health” or “attainment of full health potential” • Most stakeholders mention “societal efforts” or “social conditions” to expand on the importance of social determinants of health • Most stakeholders include “absence of disparities” or “elimination of disparities in health” in their definition of health equity		*Pharma & life sciences*	“Equity in healthcare begins with ensuring access for all to life-transforming therapies. But inequity persists and disproportionately affects already underserved and Black, Indigenous, and people of color (BIPOC) communities” *– Medtronic*^40^

Ecosystem stakeholders define health equity similarly but apply its principles through the lens of their experience, remit, and tie to business objectives.

Within health equity, there are several components that are interrelated, but distinct. Health disparities and health inequities, for example, are terms that are often used interchangeably, but have different implications. Health disparities are differences in health outcomes or indicators among different groups that are observable and measurable, such as mortality, morbidity, life expectancy, disability, quality of life, access to care, and health behaviors. Health inequities, on the other hand, are a subset of health disparities that are avoidable differences resulting from the systematic and uneven distribution of power, resources, and opportunities that affect the health of different groups. They are normative and value-based judgments that require ethical and political considerations, as well as empirical evidence, to determine what constitutes an equitable distribution of health.^41^^,^^42^

Different frameworks exist to conceptualize and analyze health equity, its determinants, and its dimensions (Table 3). One of the most influential is the social determinants of health (SDH) framework developed by the US Health and Human Services (HHS) department. This framework ties directly to Healthy People 2030 goals for the US,^43^ and asserts that health is influenced by social, economic, environmental, and political factors alongside biological and behavioral factors. The SDH framework addresses multiple levels and pathways, including individual, interpersonal, community, institutional, and structural levels, and material, psychosocial, and behavioral pathways.^44^^,^^45^ It emphasizes a life course and intergenerational perspective, recognizing cumulative and long-term effects on health and the transmission of health disparities across generations.^46^^,^^47^ This framework also highlights the state’s and society’s roles in creating and maintaining social conditions that affect health and ensuring participation and empowerment of affected groups.^48^^,^^49^ Complementing the SDH framework, the social-ecological model (SEM) employs a multi-level approach to demonstrate the interaction among various factors from the individual to societal level.^50^ It is one of the most widely cited equity models, and it identifies leverage points for intervention by governments and private companies, including the physical environment, resources, and social norms.^51^

**Table 3:** Frameworks for health equity from existing literature

Source	Definition	Key concepts	References
Whitehead (1992)	Equity in health implies that ideally everyone should have a fair opportunity to attain their full health potential and that no one should be disadvantaged from achieving this potential, if it can be avoided.	Focus on fairness, removing avoidable inequalities, equal health opportunities for all.	Whitehead et al.^33^
Institute of Medicine (2003)	Health disparities are differences in the quality of health care that are not due to access-related factors or clinical needs, preferences, and appropriateness of intervention, but are linked to racial or ethnic group membership.	Focus on healthcare disparities, racial and ethnic differences in health care quality, inequities linked to group membership.	Betancourt et al.^52^
Kawachi, I., Subramanian, S. V., and Almeida-Filho, N. (2002)	Health inequalities refer to differences in health that are unnecessary, avoidable, unfair, and unjust. Health equity is about creating opportunities for all people to be as healthy as possible and addressing systemic barriers and obstacles that cause differences in health.	Distinction between health inequalities and inequities, addressing systemic barriers and obstacles.	Kawachi et al.^53^
Braveman and Gruskin (2003)	Equity in health implies that ideally everyone should have a fair opportunity to attain their full health potential, and that no one should be disadvantaged from achieving this potential if it can be avoided. Health inequities are systematic, avoidable, and unjust differences in health status or distribution of health resources between different population groups.	Focus on systematic, avoidable, and unjust differences in health, fair opportunity for all.	Braveman et al.^54^
World Health Organization (WHO) (2008)	Health equity means that everyone should have a fair opportunity to attain their full health potential, and no one should be disadvantaged from achieving this potential. Inequities are avoidable inequalities in health between groups of people within countries and between countries.	Focus on avoidable inequalities, social justice, fair opportunities for health for all.	WHO^55^
Centers for Disease Control and Prevention (CDC) (2013)	Health equity is achieved when every person has the opportunity to “attain his or her full health potential” and no one is “disadvantaged from achieving this potential because of social position or other socially determined circumstances.”	Emphasis on opportunity for full health potential, removing disadvantages linked to social circumstances.	CDC^56^
Braveman et al. (2022)	Health equity means that everyone has a fair and just opportunity to be as healthy as possible. Achieving this requires removing obstacles to health such as poverty, discrimination, and their consequences, including powerlessness and lack of access to good jobs with fair pay, quality education, and housing.	Focus on removing social determinants of health inequity, such as poverty and discrimination.	Braveman et al.^57^
Healthy People 2030 (US Department of Health and Human Services)	Health equity is the attainment of the highest level of health for all people. Achieving health equity requires valuing everyone equally with focused and ongoing societal efforts to address avoidable inequalities, historical and contemporary injustices, and the elimination of health and healthcare disparities.	Focus on achieving the highest level of health for all, addressing avoidable inequalities and injustices, valuing all individuals equally.	Healthy People^43^

However, these existing models have limitations. They often fail to fully capture the dynamic and evolving nature of health equity, which requires continuous adaptation to the changing needs and values of society. Furthermore, while these frameworks are comprehensive, they can be overly broad, making it challenging to apply them effectively to specific contexts or populations within the US. This highlights the need for a new framework that not only integrates the principles of SDH and SEM but also addresses the unique and intersecting factors affecting health equity in the US. By conducting a detailed assessment of the social determinants and their interplay, we can develop an innovative modern definition that is more actionable and context-specific, thereby advancing health equity more effectively.

### Determinants and dimensions of health equity

**Figure 1:**
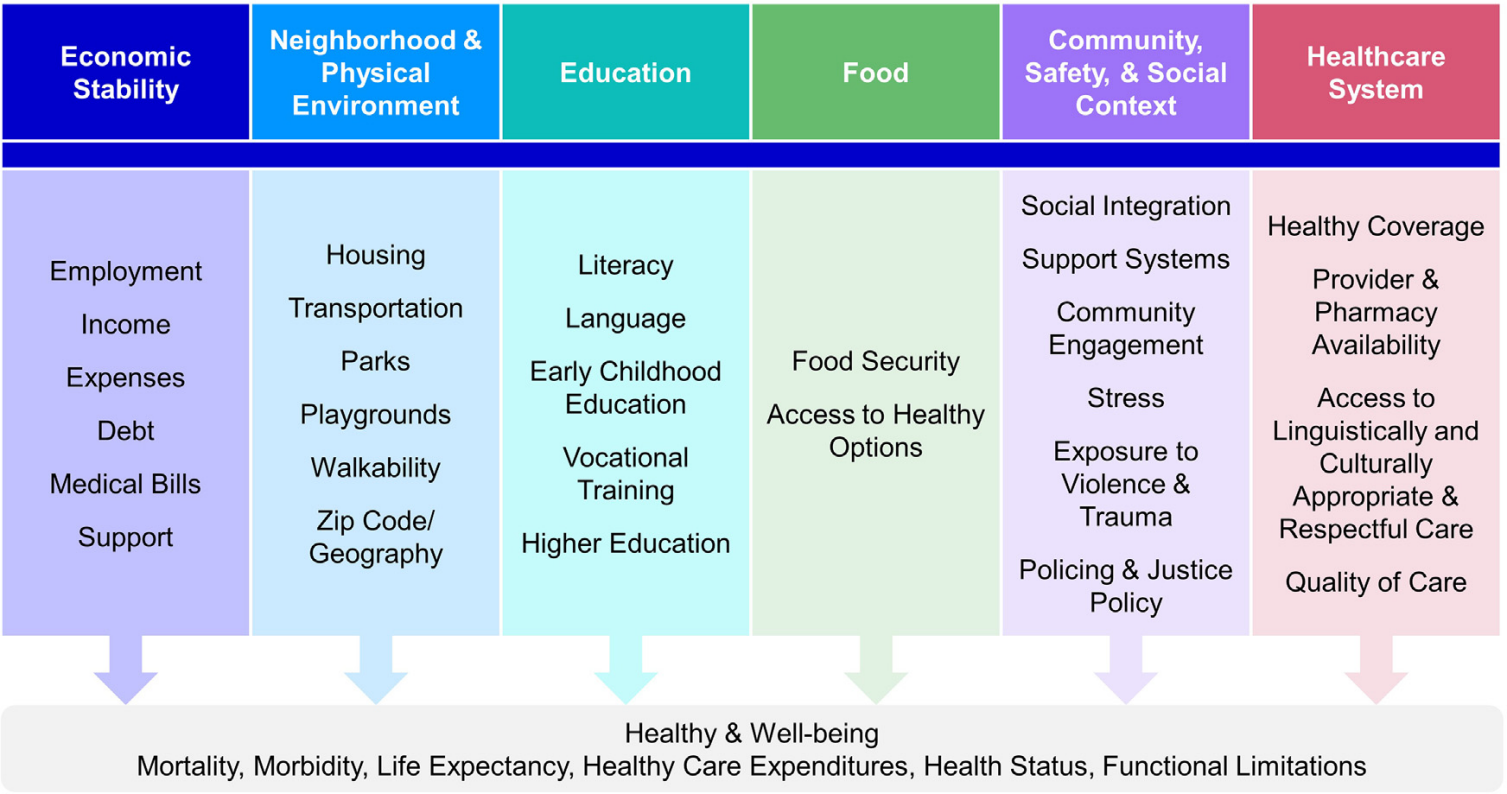
KFF’s social determinants of health Social determinants of health are the conditions in which people are born, grow, live, work and age. They include factors like socioeconomic status, education, neighborhood and physical environment, employment, and social support networks, as well as access to health care. Modified from KFF, “Beyond Health Care: The Role of Social Determinants in Promoting Health and Health Equity.” Available at: (https://www.kff.org/racial-equity-and-health-policy/issue-brief/beyond-health-care-the-role-of-social-determinants-in-promoting-health-and-health-equity/).

Developing a modern definition for health equity must involve a comprehensive understanding of its determinants and dimensions, the varied aspects that shape health disparities among different groups and individuals. The “determinants” of health equity refer to the underlying factors and conditions that distribute health and well-being unequally among different populations, and have been described in depth in the literature.^2^^,^^30^^,^^48^^,^^58^ They are often categorized into social, economic, environmental, and political factors that influence individual and group differences in health status. The Kaiser Family Foundation (KFF) has broken these factors down further to include the following: economic stability; neighborhood and physical condition; education; food; community, safety, and social context; and the health care system (Figure 1).^59^ While this does not describe the impact of these determinants on all marginalized groups, it does provide a framework for understanding the primary factors influencing health equity.

Each determinant impacts what we define as the “dimensions” of health equity: the various aspects or axes along which health equity is measured or experienced, including, but not limited to, race and ethnicity, gender, age, geography, sexual orientation, and disability. These categories are not mutually exclusive or exhaustive, and they may overlap, intersect, or change over time and across contexts. However, they provide a functional way to synthesize literature on the determinants and dimensions of health equity, and to understand the main themes that emerge.

#### Interplay of social, economic, environmental, and political factors in health equity

Current frameworks for understanding health equity typically use a static, siloed approach to its determinants and dimension. Achieving health equity, however, requires a comprehensive understanding that accounts for the complex interaction among these determinants and dimensions, and an interdisciplinary appreciation for how different sectors impact health equity. For example, examining the correlation between socioeconomic status (a determinant) and race or ethnicity (a dimension) can spotlight how systemic disparities contribute to health gaps. Likewise, gender imbalances in health can be shaped by both societal norms (a social determinant) and the availability of gender-specific healthcare services (an economic and political determinant). These deeply interconnected determinants and dimensions not only shape but are also shaped by each other, creating a dynamic and intricate landscape that significantly affects health outcomes and behaviors across various populations. Here, we explore the intricate ways in which these determinants and dimensions interact, highlighting their fluid and constantly evolving nature, and offering insights into the many ways health equity is shaped by a convergence of diverse yet interconnected factors.

##### Determinants and dimensions of health equity are interrelated and interdependent, and they influence each other in complex and dynamic ways

Social and economic determinants of health equity are intricately connected. Education, a critical social determinant, significantly influences economic opportunities, subsequently impacting health outcomes.^60^ For example, attaining higher education correlates with improved job prospects, increased income, and access to resources that can greatly enhance health outcomes.^61^ Those with a bachelor’s degree or higher are three times less likely to report poor health than those with a high school diploma only.^62^

Economic conditions also have a direct impact on social determinants like housing and healthcare access.^63^ For example, the affordability of housing, often tied to job status and income, plays a crucial role in determining an individual’s living conditions, including the safety and quality of their surroundings. Poor housing conditions are linked to various health issues, such as respiratory infections and lead poisoning.^64^ Unfortunately, nearly a quarter of the homes in the US are considered unhealthy or inadequate, and low-income families are more likely to reside in housing that poses health hazards.^65^

Environmental and policy determinants of health equity are also strongly linked. For example, access to clean air, water, safe housing, and green spaces, are greatly impacted by political decisions. Policies regulating emissions, ensuring safe drinking water, and allocating funds for urban green spaces directly influence environmental conditions affecting health.^66^^,^^67^ The Flint water crisis stands out as an example of how political neglect and environmental injustice can trigger severe public health crises, disproportionately impacting marginalized communities.^68^ In Flint, Michigan, decisions by political leaders to change the water source without proper treatment resulted in lead contamination affecting numerous residents.

Political determinants also influence the broader economic and social landscape in the pursuit of health equity.^69^ Healthcare policies, like the Affordable Care Act (ACA) in the United States, exemplify how political actions can directly tackle economic barriers to healthcare access.^70^^,^^71^ The ACA notably decreased the uninsured rate among low-income individuals and minorities, according to HHS, through Medicaid expansion and subsidies for private insurance.

##### Determinants and dimensions of health equity are not fixed or static, but rather fluid and dynamic, and they change over time and across contexts

Health equity determinants are not rigid; they are fluid and ever-changing, varying across time and contexts. This adaptation mirrors shifts in societal norms, technological progress, environmental approaches, economic frameworks, and political priorities, all deeply influencing health outcomes and discrepancies.

The rise of technology and the internet, for example, has transformed how people and communities engage, find information, and maintain their well-being.^72^ Digital platforms and social media have modernized communication, granting swift access to health resources. This shift carries significant implications for health literacy, allowing more individuals to learn about health practices. Yet, it also poses challenges due to the digital divide, widening disparities in technology access and impacting health equity. For example, older adults or low-income households may face obstacles in benefiting from digital health tools if they lack reliable internet access.^73^

On the environmental front, the addition of green spaces in urban areas showcases the positive impact changing environmental determinants can have on health.^74^ Urban green spaces not only enhance air quality but also provide opportunities for physical activity and social interaction, leading to improved cardiovascular and mental health outcomes. The shift toward greener urban landscapes is a strategic response to the challenges of urbanization and environmental degradation.

Economic determinants also undergo dynamic shifts, evident in the shift from manufacturing to service-based economies in various regions. This restructuring has changed occupational health risks, leading to increased workplace stress and the rise of chronic diseases linked to sedentary work settings.^75^ These economic changes underscore the necessity for adaptable public health strategies that tackle emerging health risks and inequalities amid evolving labor markets and job structures.

Political determinants, including shifts in policy and healthcare systems, are pivotal in shaping health equity.^76^ The establishment of universal healthcare in certain nations has fundamentally reshaped care accessibility, showcasing how policy adjustments can directly impact health results. Universal healthcare systems strive to diminish health disparities by ensuring all individuals, irrespective of economic status or background, can access essential healthcare services.^77^ This healthcare policy approach mirrors an evolving comprehension of health equity.

##### Determinants and dimensions of health equity are not homogenous or monolithic, but rather heterogeneous and diverse, and they vary and differ among and within different groups and individuals

The intricate ways in which SDH impact diverse marginalized groups highlight the complexity of achieving health equity. These determinants, while consistently present, manifest uniquely across communities, influenced by factors such as ethnicity, location, income, and gender.

Ethnic and cultural factors, for example, play a significant role in healthcare access. Take non-English speaking immigrants in the US, who may encounter obstacles in accessing healthcare due to language barriers and a lack of culturally tailored care.^78^ These challenges not only hinder their ability to seek medical assistance but also impact communication with healthcare providers, resulting in poorer health outcomes. Initiatives such as interpreter services and culturally sensitive health programs are vital in addressing these disparities.^79^

The difference between urban and rural environments shows how environmental determinants can impact health outcomes based on location.^80^ Rural areas with a large presence of agricultural industries may face health risks from farming activities like pesticide exposure,^81^ a health threat less commonly found in cities. On the other hand, city residents might encounter industrial pollutants, increasing the risk of pollution-related health outcomes.^82^ Tackling these environmental challenges needs tailored strategies considering the unique traits of each setting.

Economic gaps within and between groups also underscore the diverse dimensions of health determinants. Discrepancies between the affluent and the disadvantaged result in notable health inequities, as those with higher incomes often experience better health.^83^ This difference arises from unequal access to vital resources like top-tier healthcare, nutritious food, and secure housing. In contrast, lower-income groups are at higher risk of chronic stress, poor nutrition, and limited healthcare, leading to inferior health outcomes. Addressing these disparities involves implementing policies to reduce income inequality and enhance healthcare and living conditions for all socioeconomic groups.

Political determinants also showcase variations in impact across demographic groups. Reproductive health policies, for instance, have a disproportionate effect on women’s health outcomes. The provision of services like family planning, prenatal care, and safe childbirth options plays a direct role in influencing women’s health and well-being.^84^ Gender-sensitive health policies that acknowledge and tackle these specific dimensions are crucial for enhancing health outcomes for women and ensuring fair access to healthcare services.

Overall, the exploration of health equity through the lens of its social, economic, environmental, and political determinants and dimensions reveals a complex and interconnected landscape. These factors, each with their unique influence, collectively shape the health outcomes of individuals and communities in a dynamic and ever-changing manner. This analysis underscores the importance of recognizing the fluidity and diversity inherent in these determinants. As they vary significantly across different groups and contexts, it becomes clear that a one-size-fits-all approach to health equity is ineffective. Instead, tailored strategies that acknowledge the unique interplay of these determinants in specific contexts are crucial. This nuanced understanding paves the way for more effective interventions and policies that can address the multifaceted nature of health inequities, ultimately leading to more equitable health outcomes for all individuals, regardless of their social, economic, environmental, or political circumstances.

### Interdisciplinary approaches to health equity

Defining, measuring, and analyzing health equity are crucial steps, yet insufficient on their own to achieve it. To truly advance health equity, effective and innovative interventions must be developed and implemented following a modern framework for health equity. These interventions should aim to reduce or eliminate health disparities among diverse groups, promoting the well-being of all. However, the strategies for advancing health equity are multifaceted, operating at various levels and pathways, necessitating a holistic approach to designing and implementing these interventions.

**Figure 2:**
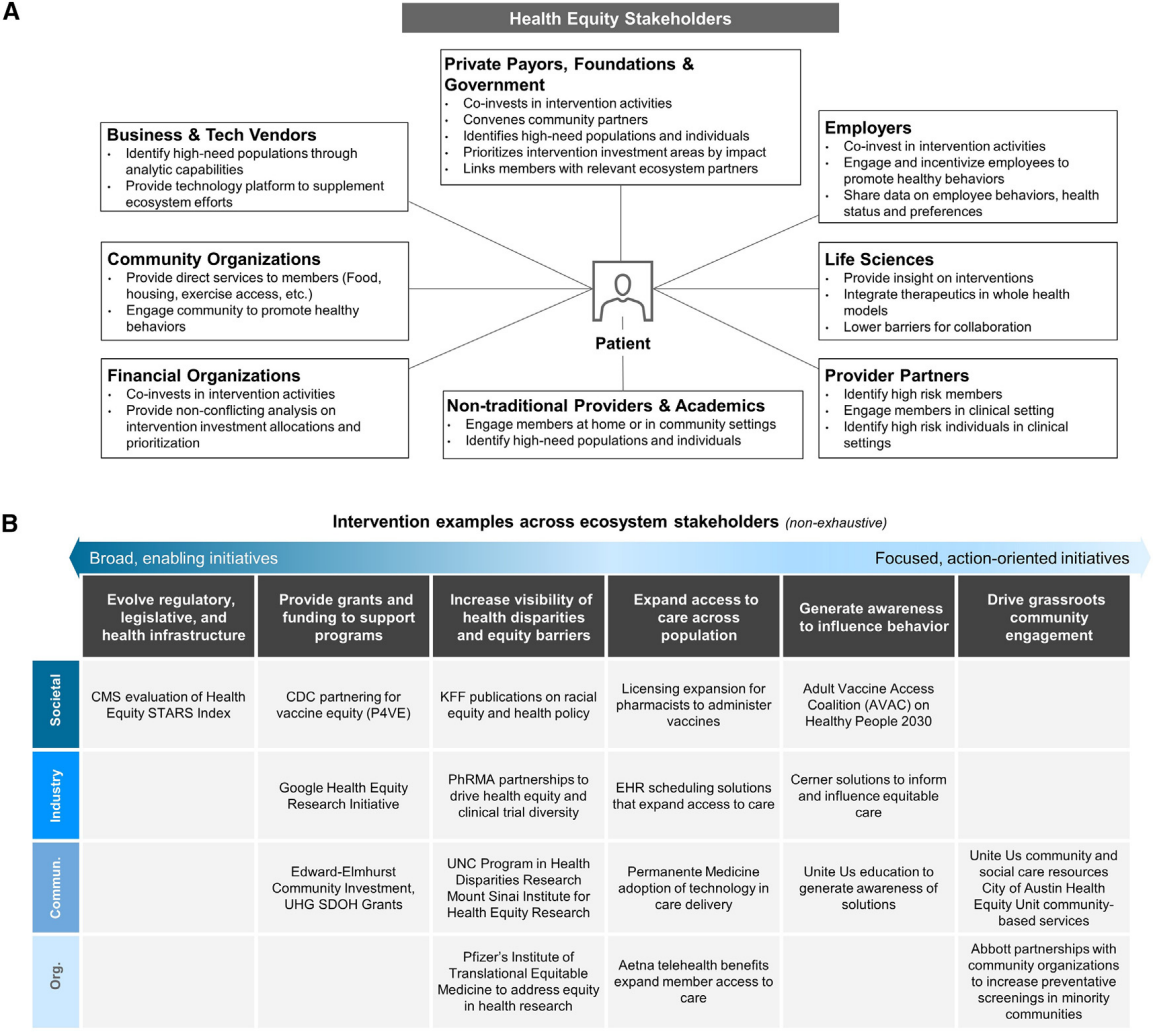
The health equity landscape (A) A diagram detailing the various health equity stakeholders and their roles, including business and tech vendors, community organizations, financial organizations, private payors, government, employers, life sciences, provider partners, and non-traditional providers and academics. (B) A table presenting intervention examples across ecosystem stakeholders, categorized by broad, enabling initiatives and focused, action-oriented initiatives. The table highlights efforts at societal, industry, community, and organizational levels. AVAC, Adult Vaccine Access Coalition; CDC, Centers for Disease Control and Prevention; CMS, Centers for Medicare & Medicaid Services; Comm., Community; EHR, Electronic Health Record; KFF, Kaiser Family Foundation; Org., Organization; P4VE, Partnering for Vaccine Equity; PhRMA, Pharmaceutical Research and Manufacturers of America; PRHE, Permanente Medicine (likely referring to Kaiser Permanente); SDOH, Social Determinants of Health; UHG, United Health Group; UNC, University of North Carolina.

Any approach for advancing health equity will require engaging relevant stakeholders, which extend beyond traditional healthcare players (Figure 2A). Such non-traditional stakeholders include community organizations, employers, business and technology vendors, and financial organizations, all of which play crucial roles in this landscape. Due to the complex, interrelated, and dynamic nature of the determinants and dimensions of health, these stakeholders need to cross the boundaries of their industries and work together to develop interdisciplinary and comprehensive solutions that address barriers to health equity. To do so, there needs to be the exchange of values and ideas between industries, consistent analysis of new data, and the development of risk mitigation and control strategies.

The increasing body of evidence on health equity emphasizes a consensus on the effectiveness of evidence-based, participatory, and holistic strategies.^85^^,^^86^^,^^87^^,^^88^ Examples of such interventions, across the health equity landscape and ranging from broad, enabling initiatives to focused, action-oriented strategies, are provided in Figure 2B. Additionally, the US has been developing strategies like the Center for Disease Control and Prevention’s (CDC’s) initiatives aimed at tackling the disproportionate impact of COVID-19 on historically marginalized groups such as Native American, Hispanic, and Black American communities.^89^ These efforts involved utilizing culturally sensitive health communication methods and fostering community-academic collaborations. Programs like Better Together REACH (Racial and Ethnic Approaches to Community Health) and Project ECHO (Extension for Community Healthcare Outcomes) were pivotal in providing consistent support to Hispanic populations in Pennsylvania by adapting existing chronic disease initiatives to address pandemic challenges.^90^^,^^91^ CDC-funded projects have also made significant strides in addressing health inequalities.^92^ These endeavors encompass reducing high-risk sexual conduct among black men who have sex with men, the success of the Vaccines for Children (VFC) Program in upholding enduring immunization coverage with sustained political backing, and evidence-based road safety measures in American Indian/Alaska Native tribal communities that have notably decreased motor vehicle-related injuries and fatalities.

The landscape is also evolving to include the lens of other disciplines to better understand, study, and intervene effectively on health equity. One example includes using behavioral economics to understand underlying drivers of vaccine attitudes that lead to low uptake in marginalized groups. This includes analyzing the impact of COVID-19 vaccine attitudes on the rate of other vaccinations, such as those for influenza.^93^ There has also been a push to layer in the science of genetic diversity to better understand the link between genetic diversity and health disparities and help to identify opportunities to influence health by addressing these underlying factors.^94^

Digital technology is also poised to play a crucial role in advancing health equity by providing innovative tools and platforms that can address systemic disparities in healthcare.^95^ Artificial Intelligence (AI) and machine learning can analyze large datasets (e.g., omics data) to identify patterns and disparities, enabling the development of targeted interventions for underserved populations.^96^ Digital health platforms can improve access to care through telemedicine, mobile health applications, and remote monitoring, ensuring that even those in remote or low-resource settings receive timely and effective care.^97^ For example, virtual assistants can enhance medication adherence and patient monitoring.^98^ Another potential use case is the implementation of AI tools to harmonize clinical recommendations, ensuring they are equitable across diverse patient populations. These tools could help identify biases in clinical guidelines and adjust them to account for varying needs and conditions across different demographic groups.

The approaches highlighted above take a comprehensive view, not only addressing biological and behavioral health disparities but also examining the wider social, economic, environmental, and political factors at play. By respecting the diversity and complexity of affected groups’ needs and preferences, this inclusive approach contributes to enhancing health outcomes, quality of life, well-being, and the protection of human rights for individuals facing health disparities.

### A modern framework to define health equity

**Figure 3:**
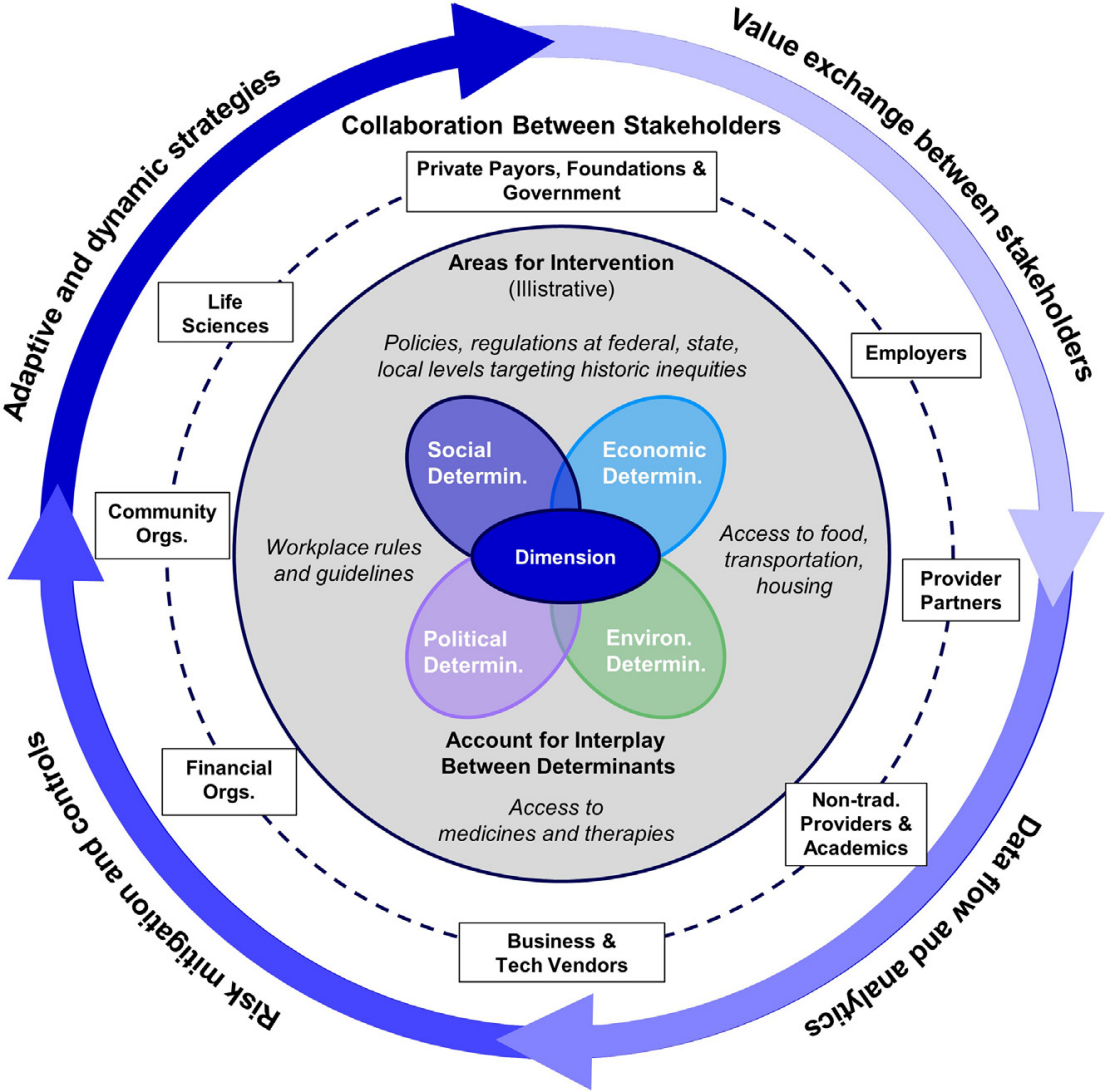
A modern framework for defining health equity A modern definition of health equity is one that must be context-specific for each dimension (race, gender, geography, etc.) and accounts for the interplay between determinants of health equity, highlights areas for intervention, and encourages collaboration between stakeholders. It must also account for the changing landscape by encouraging an iterative process that involves the exchange of values between stakeholders, consistent data flow and the analysis of that data, and the implementation of risk mitigation and control strategies, all culminating in adaptive and dynamic strategies for ensuring health equity. Determin., determinants; Orgs., organizations; Non-trad., non-traditional.

Drawing from an assessment of social determinants and dimensions of health and the evolving landscape, we propose a modern framework for defining health equity that emphasizes proactive, context-specific strategies addressing the dynamic and intersecting social, economic, environmental, and political determinants of health (Figure 3). Here, health equity in the US is defined as the pursuit of fairness and justice in health, where all individuals have equitable access to resources, opportunities, and healthcare necessary to achieve their full health potential, irrespective of their socioeconomic, racial, or geographical backgrounds. This definition acknowledges the historical, systemic, and dynamic factors contributing to health disparities and emphasizes a proactive approach to dismantling these barriers through policy, practice, and community engagement.

This framework for defining health equity departs from traditional approaches by acknowledging the fluid and evolving nature of these determinants, such as social, economic, environmental, and political factors, which are not fixed but rather dynamic and interconnected. By recognizing that these determinants change over time and across different contexts, this framework necessitates adaptive strategies that are responsive to changing landscapes, such as shifts in technology, policy, economic conditions, and environmental factors. Unlike conventional frameworks, which often focus on describing health disparities and inequalities without providing clear guidance for action, this framework moves beyond a descriptive focus to a prescriptive one. It emphasizes proactive and actionable strategies that are tailored to the unique needs of specific contexts and populations, taking into account intersectionality, historical and systemic barriers, and the role of various stakeholders across multiple sectors. This comprehensive, context-specific approach encourages continuous adaptation, collaboration, and interdisciplinary efforts, ensuring that interventions are both equitable and effective in addressing the root causes of health disparities.

In applying this modern definition, health equity initiatives in the US should focus on adaptive, inclusive strategies that respond to the evolving determinants of health. By doing so, we can create more effective interventions that genuinely promote equitable health outcomes, ensuring every individual, regardless of their background or circumstances, has the opportunity to attain optimal health.

Research will need to form the basis of any health equity intervention. It is important to conduct and disseminate research that advances knowledge and evidence on health equity and its determinants and dimensions. This research should inform and influence health equity policies and practices. Utilizing appropriate and rigorous methods and indicators to define and measure health equity and its impacts is essential. Incorporating and respecting the perspectives and experiences of those affected by health disparities in research processes is key. Their participation and benefit should be ensured throughout the research process and in the resulting products.

Data collected and analyzed in health equity research will need to form the basis of new policy. It is essential to develop and implement policies and programs that address the social, economic, environmental, and political determinants of health. These policies should also focus on the underlying causes of health disparities and inequities, promoting the right to health and human rights for all. Policy initiatives need to be evidence-based, participatory, transparent, accountable, and responsive to the needs and preferences of the people and groups affected by health disparities. Monitoring and evaluating the impacts and outcomes of these policies will be crucial to making necessary adjustments and improvements.

Developing and implementing policies will need the buy-in and participation from all stakeholders. This means enhancing the capacity and competence of the health and other sectors and stakeholders and enabling a better understanding of health equity and its determinants and dimensions. Adopting and applying the principles and standards of health equity and human rights in everyday life and work will be vital. Collaboration and coordination among sectors and stakeholders are encouraged to create platforms for dialogue, exchange, and learning on health equity issues and solutions. Building trust with, and empowering, those affected by health disparities is also crucial, ensuring their involvement and leadership in designing, delivering, and evaluating health services and interventions.

### Conclusion

The pursuit of health equity presents a formidable challenge and a critical goal for the US, a fact made clearer by the inequity of health outcomes during the COVID-19 pandemic.^30^ By examining the intricate determinants of health disparities encompassing social, economic, environmental, and political factors, the need for embracing a comprehensive and participatory approach to bridge these gaps becomes clear. Achieving health equity goes beyond medical interventions; rather, it necessitates a collective societal commitment involving policymakers, healthcare professionals, researchers, and communities.

The recommendations provided in this context for policy, practice, and research, while not exhaustive, signify significant progress toward dismantling the systemic barriers perpetuating health inequities. As members of an interconnected society, advocating for and implementing these strategies with steadfast dedication to justice, empathy, and equity is our shared responsibility. We call on all stakeholders to intensify their efforts in advancing health equity, emphasizing the importance of coordinated and sustained engagement across all sectors of society.

The pursuit of this objective requires a unified and enduring commitment from all segments of society. It is a mission that addresses both current disparities and proactively works to mitigate future inequities. Therefore, it is crucial that we combine our collective efforts, knowledge, and determination to shape a healthier, more equitable future for all individuals, irrespective of their socioeconomic, racial, or cultural backgrounds. Attaining health equity is not optional but essential for the progress of our society and the well-being of future generations.

## Acknowledgments

This work was sponsored by 10.13039/100004319Pfizer.

## Author contributions

K.R.P. developed the outline, researched sources, and drafted and edited the manuscript. K.R.P., M.B., A.H., and C.H.J. provided strategic input, as well as drafting and editing support. C.H.J. conceptualized the publication and provided strategic oversight and editing.

## Declaration of interests

C.H.J. report that they are employees of Pfizer Inc. and may hold stock or stock options in the company. This work was sponsored by Pfizer. M.B. and A.H. are employees of Bulmore Consulting, which was a paid consultant to Pfizer in connection with the development of this manuscript.

## Declaration of generative Artificial Intelligence and AI-assisted technologies in the writing process

During the preparation of this work, the authors used Microsoft Copilot to summarize peer review comments and suggest ideas for edits. After using this tool, the authors reviewed and edited the manuscript as needed and take full responsibility for the content of the published article.
